# The impact of awareness on epidemic spreading in networks

**DOI:** 10.1063/1.3673573

**Published:** 2012-01-03

**Authors:** Qingchu Wu, Xinchu Fu, Michael Small, Xin-Jian Xu

**Affiliations:** 1 College of Mathematics and Information Science, Jiangxi Normal University, Nanchang 330022, China; 2 Department of Mathematics, Shanghai University, Shanghai 200444, China; 3 Institute of Systems Science, Shanghai University, Shanghai 200444, China; 4 School of Mathematics and Statistics, University of Western Australia, Crawley, WA 6009, Australia; 5 Department of Electronic and Information Engineering, Hong Kong Polytechnic University, Hung Hom, Kowloon, Hong Kong

## Abstract

We explore the impact of awareness on epidemic spreading through a population represented by a scale-free network. Using a network mean-field approach, a mathematical model for epidemic spreading with awareness reactions is proposed and analyzed. We focus on the role of three forms of awareness including local, global, and contact awareness. By theoretical analysis and simulation, we show that the global awareness cannot decrease the likelihood of an epidemic outbreak while both the local awareness and the contact awareness can. Also, the influence degree of the local awareness on disease dynamics is closely related with the contact awareness.

The interplay between awareness and epidemic dynamics in networks has recently achieved much attention. The human responses to disease outbreaks can result in the reduction of susceptibility to infection, which in turn, can affect epidemic dynamics. So an epidemic model should include such factors. This issue has been studied from the perspective of awareness reactions. However, the impact of individual awareness is not entirely understood thus far because of its variety and complexity. In this work, we build a continuous mean-field (MF) model to study the impact of the three forms of awareness on the epidemic spreading in a finite scale-free (SF) network: contact awareness that increases with individual contact number; local awareness that increases with the fraction of infected contacts; and global awareness that increases with the overall disease prevalence. Theoretical analysis and simulation shows that the effect of these different types of awareness can be clearly classified. Both the contact awareness and the local awareness can raise the epidemic threshold, while the global awareness can only decrease the epidemic prevalence. These results also tell us that individual awareness contributes toward the inhibition of epidemic transmission.

## I. INTRODUCTION

During the outbreak of influenza A (H1N1) in 2009, the effect on human behaviors (such as staying at home and wearing surgical face masks) not only due to public measures but also due to individual responses was widely documented.^1^ When aware of an infectious disease outbreak, people will sometimes change their behavior in order to reduce the risk of infection.^2^ Interestingly, the change of individual behaviors in the presence of an infectious pathogen also has an effect on the epidemic spreading.

Recently, there has been growing interest in investigating ways to model aspects of human responses to disease outbreaks in epidemiological models including network epidemic models^3–5^ and non-network epidemic models.^6–8^ In general, individual behaviors in the presence of an infectious pathogen respond to the information obtained from the general circumstances. Following Funk *et al.*,^4^ such information may come from the social or spatial neighborhood, which is called local (available) information. Another source of information is from the media (e.g., the information published by public health authorities), called global (available) information.

In modeling the effect of human behavior on epidemic transmission, apart from the sources of information described above, the effect of behavioral changes is also important. In light of the classification method proposed in Ref. 4, the behavioral changes must affect either: (1) the disease state (e.g., healthy state or vaccinated state) of the individual; (2) the infection rate^9–11^ or the recovery rate (may including the contact rate^7^); or (4) the contact network structure relevant for the spread of disease.^3,12–15^ In this work, we only consider the effect of individual responses on the infection rate. So, we suppose that the network structure is considered not to depend on the infection level.^11^ Although this restriction may limit the realism of our model, it allows us to focus on the information effect for a mild infectious disease, e.g., flu. It is only under an extremely serious epidemic situation that the measures of strong quarantine or isolation would be implemented,^16^ which will induce changes in the social network.

For simplicity, we call the change of individual behavior to infection *individual awareness*.^6,9^ Awareness causes individuals to keep social distance^9^ (by wearing protective masks, vaccination, or more creative precautions), which (potentially) results in the reduction of individual susceptibility. The study of this issue may be classified into the two kinds of perspectives

(1) The spread of awareness (or the information transmission), which assumes that the information (generally from an infectious node) undergoes a generation process and a transmission process from individual to individual. In order to study the effect of information transmission, two separate networks can be used for modeling the epidemic spreading and the information spreading, respectively.^9^ Another approach is to classify a population with respect to information.^5,6,8^ In general, the local spread of awareness can stop a disease from spreading,^5,9^ while the global transmission of information can only decrease the prevalence.^5^
(2) The reaction of awareness (or the risk perception), which means that an individual promptly obtains relatively accurate information from the current circumstances and responses to the epidemics. In the study of this, the effect of risk perception can be expressed by a function of information. In Refs. 10 and 11, an exponential function of local information is used to study the transition of the level of precautionary measures, where the network structure has important impact on the existence of a value of perception that stops the epidemics.^10^

In the present work, we investigate this issue from the second perspective in the heterogenous SF network, which exhibits a broad degree distribution.^17^ Different from the previous work (see Refs. 10 and 11), we consider many types of information, which include both local information and global information. One advantage of this approach is that it allows us to study the difference between local information and global information.^4^ Besides these prevalence-based information, we also consider one kind of belief-based information^4^ which is related to individual nodes’ contact numbers called contact information. This accounts for awareness of a higher risk when a node possesses a larger contact number. The study of multiple information complies with the variety and complexity of information in reality.^5^ The assumption of the static network allows us to focus on the impact of such multiple information/awareness on the epidemic spreading.

The rest of this paper is organized as follows: In Sec. II, we propose an SIS (susceptible-infected-susceptible) model with awareness reactions; then in Sec. III, we analyze the conditions for epidemic spreading and determine the epidemic threshold; in Sec. IV, we present numerical simulations and compare these to the theoretical model and investigate the impact of both the local awareness and the global awareness on the epidemic prevalence (i.e., the final epidemic size); and finally, in Sec. V, we conclude the paper and give some discussion.

## II. THE MODEL

The epidemics we study spread on scale-free networks of *N* nodes^17–19^ with degree *k* distributed according to *P*(*k*), where *P*(*k*) is the fraction of nodes with connectivity *k*. Since we restrict our attention to the impact of multiple awareness (or information) on the epidemic spreading, it is assumed that the connectivity of nodes in networks is uncorrelated, which make the following discussion simpler. The infection rate, the rate that susceptible individuals are infected by an infectious neighbor, is always related to susceptibility and infectivity of individuals.^20–22^ To characterize this, we use the two concepts proposed by Olinky and Stone,^23^ the admission rate (characterizing susceptibility) and the transmission rate (characterizing infectivity). The admission rate *A*_*i*_ is the rate that susceptible node *i* would actually admit an infection through an edge connected to an infected node. The transmission rate *T*_*i*_ is the rate that infected node *i* would actually transmit an infection through an edge connected to a susceptible node.

If we denote by *q*_*ij*_, the infection rate along the edge between *i* and *j*, then, we have^23,24^

$$q_{\mathrm{ij}}=\left\{ \begin{array}{cc} A_{i}T_{j}, & i\mathrm{is}\mathrm{susceptible}\mathrm{and}j\mathrm{is}\mathrm{infectious};\\ T_{i}A_{j}, & i\mathrm{is}\mathrm{infectious}\mathrm{and}j\mathrm{is}\mathrm{susceptible};\\ 0, & \mathrm{otherwise}. \end{array}\right.$$ (1)

In cases of no awareness, it is usually assumed *T*_*i*_ = λ and *A*_*i*_ = 1. Here, we still assume that *T*_*i*_ = λ, but the admission rate ***A***_*i*_ is coupled with individual awareness or information.

Considering the complexity of individual awareness or information,^5^ we introduce three forms of awareness. The first is dependent of individual contact number (i.e., contact information). In social networks, the contact number can be denoted by the node degree. Intuitively, the larger the contact number, the higher the risk of being infected. So the reaction to contact information (this should be belief-based information^4^) is called the *contact awareness*. The contact awareness, therefore, can reduce individual susceptibility and affect the admission rate, represented by ψ(*k*_*i*_) as a multiplicative factor^9^ in the expression for *A*_*i*_. Obviously, ψ(*x*) is a decreasing function of *x*.

On the other hand, the conscious behavior of individuals will also change in reaction to epidemic information and affect the epidemic spreading in turn. Such information includes both the local infection density $\varrho_{i}$ in node *i*’s vicinity/neighborhood (i.e., the local information^10,11^) and the global infection density ρ in a whole community^2^ (i.e., the global information). Hence, the other two kinds of awareness are called *local awareness* and *global awareness* corresponding to the local information and the global information, respectively.

Similar to the contact awareness, both the local awareness and the global awareness may impact the admission rate with two multiplicative factors. Herein, we first consider a general scenario. If we denote the epidemic information by *x*, then $x=\varrho_{i}$ for the local information and *x* = ρ for the global information. We introduce a function of *x*, φ(*x*), as a multiplicative factor of *A*_*i*_ to characterize the impact of information on the admission rate of node *i*, which satisfies 0 ≤ φ(*x*) ≤ 1, φ(0) = 1, and φ′(*x*) < 0.

In Bagnoli *et al.*,^10^ $\varphi (x)=e^{-{\mathrm{Jx}}^{\theta }}$. Here, *J* stands for the level of precaution measures adopted and 0 ≤ θ ≤ 1 denotes the use of special prophylaxis. And *x* cannot only represent the local information (denoted by *x*_1_) but also the global information (denoted by *x*_2_). So, in the literature,^10^ $A_{i}=\varphi (x_{1})\varphi (x_{2})=\exp[-({\mathrm{Jx}}_{1}^{\alpha }+x_{2})]$. Although this form is interesting and frequently used,^7^ the authors obtained the epidemic threshold only for a special case: *x*_2_ = constant.^10^ In this work, we take another frequently used form φ(*x*) = 1 − *cx* where constant *c* is referred as the impact strength of the epidemic information on the admission rate and 0 ≤ *c* ≤ 1.

Based on the above analysis, we have a specific expression of *A*_*i*_ for node *i* (here, ***A***_*i*_ has been regarded as a function of the entire network) as follows:

$$A_{i}=\psi (k_{i})\varphi (x_{1})\varphi (x_{2})=\psi (k_{i})(1-\alpha \varrho_{i})(1-\beta \rho ).$$ (2)

where *c* = α for the local awareness and *c* = β for the global awareness, respectively. In other words,

$$T_{i}=\lambda ,A_{i}=\psi (k_{i})\left(1-\alpha \frac{k_{\inf}^{i}}{k_{i}}\right)[1-\beta \rho (t)],$$

where $k_{\inf}^{i}$ is the total number of node *i*’s infected neighbors. We further suppose that the definition of (*T*_*i*_, *A*_*i*_) (2) holds for all nodes in the network. That is, all nodes can uniformly change their behavior in response to infection, which may be regarded as a kind of statistically synchronized behavior^25^ and can be easily revised for more realistic cases. For example, we can assume that Eq. (2) holds for a portion (but not all) of the nodes in the network, which has been investigated from the perceptive of information transmission.^5,6^

It is worth noting that Olinky and Stone^23^ analyzed the case *T*_*i*_ = *T*(*k*_*i*_) and *A*_*i*_ = *A*(*k*_*i*_) and found that such degree-correlated infection rates can decrease the potential of an epidemic outbreak. In our work, *A*_*i*_ is dynamical, not only dependent of connectivity structures (this point is not included in the work^10,11^) but also coupled with epidemic information.

In this context, we use SIS dynamics to investigate the effect of awareness. In our model, each individual exists only in two discrete states: S-susceptible and I-infected. At each time step, each susceptible (healthy) node *i* is infected with rate *q*_*ij*_ if it is contacted by one infected individual *j*; and an infected node is cured and becomes susceptible again with rate γ (i.e., the recovery rate).

Let Θ(*t*) be the probability of a randomly selected link pointing to an infected individual and ρ_*k*_(*t*) be the infection density among nodes with degree *k* at time step *t*, then, we have^26^

$$\Theta (t)=\frac{\sum_{k}\mathrm{kP}(k)\rho_{k}(t)}{\sum_{k}\mathrm{kP}(k)}=\frac{\sum_{k}\mathrm{kP}(k)\rho_{k}(t)}{〈k〉}.$$ (3)

The probability that a node with degree *k* has exactly *s* infected neighbors is given by the binomial distribution^27^

$$B(k,s)=\left(\begin{array}{c} k\\ s \end{array}\right){\left[\Theta (t)\right]}^{s}{\left[1-\Theta (t)\right]}^{k-s}.$$ (4)

If a susceptible node with degree *k* has exactly *s* (*s* ≤ *k*) infected neighbors, then the probability of infection is $w(s):=1-{\left\{1-\lambda \psi (k)(1-\alpha \frac{s}{k})[1-\beta \rho (t)]\right\}}^{s}$, where we adopt the nonlinear contagion scheme.^27^ Taking the expectation of *w*(*s*) with respect to the above defined binomial distribution indicates that a susceptible node with degree *k* is infected with probability

$$\mathrm{Prob}(S\to I)≅E[w(s)]=1-\sum_{s}B(k,s)\times {\left\{1-\lambda \psi (k)(1-\frac{\alpha s}{k})[1-\beta \rho (t)]\right\}}^{s}.$$ (5)

Then, the discrete-time epidemic process can be described as follows:

$$\rho_{k}(t+1)=(1-\gamma )\rho_{k}(t)+[1-\rho_{k}(t)]E[w(s)].$$ (6)

Let us consider the epidemic spreading as a continuous-time process^28^ and assume that in the infinitesimal interval (*t, t* + *h*] (Ref. 29), a susceptible individual is infected by an infectious one with probability $\lambda h\psi (k)(1-\alpha \frac{s}{k})[1-\beta \rho (t)]+o(h)$, and an infected individual can recover to be healthy with probability γ*h* + *o*(*h*). Then, we have

$$\begin{array}{c} \rho_{k}(t+h)-\rho_{k}(t)=-\gamma h\rho_{k}+o(h)+(1-\rho_{k}){\left\{1-\sum_{s}B(k,s)[1-\lambda h\psi (k)\left(1-\frac{\alpha s}{k}\right)(1-\beta \rho )+o(h)\right]}^{s}\}. \end{array}$$ (7)

Furthermore, we have

$$\begin{array}{c} \rho_{k}(t+h)-\rho_{k}(t)=-\gamma h\rho_{k}+\{1-\sum_{s}B(k,s)H^{s}(s,k)\}\times (1-\rho_{k})+o(h), \end{array}$$ (8)

where *H*(*s,k*) = 1 − λ*h*ψ(*k*)(1 − α*s*/*k*)(1 − βρ). The detailed proof for Eq. (8) can be found in Appendix. Notice that

$$\begin{array}{c} \underset{h\to 0}{\text{lim}}\frac{1-\sum_{s}B(k,s)H^{s}(s,k)}{h}=\underset{h\to 0}{\text{lim}}\sum_{s}B(k,s){\mathrm{sH}}^{s-1}(s,k)\lambda \psi (k)(1-\alpha s/k)(1-\beta \rho )=\lambda \psi (k)(1-\beta \rho )\sum_{s}B(k,s)s(1-\alpha s/k)=\lambda \psi (k)(1-\beta \rho )\{E[s]-\frac{\alpha }{k}E[s^{2}]\}=\lambda k\psi (k)(1-\alpha \Theta )(1-\beta \rho )\Theta -\lambda \alpha \psi (k)(1-\beta \rho )\Theta (1-\Theta ). \end{array}$$

Thus, dividing by *h* and letting *h* → 0 in Eq. (8), one can get the following mean-field rate equations:^26^

$$\begin{array}{c} \frac{d}{\mathrm{dt}}\rho_{k}(t)=-\gamma \rho_{k}+\lambda k\psi (k)(1-\rho_{k})\Theta (1-\alpha \Theta )(1-\beta \rho )-\lambda \alpha \psi (k)(1-\rho_{k})(1-\beta \rho )\Theta (1-\Theta ). \end{array}$$ (9)

In the derivation of Eq. (9), the first/second moment of the binomial distribution Eq. (4) E[s]=kΘ and $E[s^{2}]=k^{2}\Theta^{2}+k\Theta -k\Theta^{2}$ is used. The fraction of infected nodes over the entire network is such that^26^

$$\rho (t)=\sum_{k}P(k)\rho_{k}(t).$$ (10)

It is noticed that without loss of generality, we can set γ = 1 in model (9). Hence, unless otherwise specified, we assume the recovery rate γ = 1.

It is interesting to consider a special form in model (9). When α = β = 0 and ψ(*k*) = 1, the model is

$$\frac{d}{\mathrm{dt}}\rho_{k}(t)=-\rho_{k}+\lambda k(1-\rho_{k})\Theta .$$

This model is just the networked SIS model proposed by Pastor-Satorrás and Vespignani.^26^

## III. EPIDEMIC THRESHOLD

A main feature of the infection which we want to estimate is the epidemic threshold for transmission rate λ_*c*_. If λ ≤ λ_*c*_, the modeled disease dies out, otherwise, the disease spreads. The epidemic threshold is actually equivalent to a critical point in a disequilibrium phase transition.^26^ A widely used method to analyze the epidemic threshold is to establish the existence of the positive stationary state: as was introduced by Pastor-Satorras and Vespignani.^26,30^ However, this approach seems to be not suitable for our model. Herein, we make use of another approach, i.e., to determine the local stability of the infection-free equilibrium, which is similar to deriving the basic reproduction number in mixed populations.^31,32^ For the sake of the following analysis, we first present a lemma.

Lemma 1: For the real matrix *A* = [*a*_*ij*_] ∈ *R*^*n*×*n*^ where *a*_*ij*_ = δ_*ij*_υ_*i*_ + σ_*i*_*l*_*j*_ and δ_*ij*_ is the Kronecker symbol, we have that the determinant of *A* is such that

$$\begin{array}{c} \text{det}[A]=\upsilon_{1}\upsilon_{2}\ldots \upsilon_{n}+\sigma_{1}l_{1}\upsilon_{2}\ldots \upsilon_{n}+\upsilon_{1}\sigma_{2}l_{2}\upsilon_{3}\ldots \upsilon_{n}+\ldots +\upsilon_{1}\upsilon_{2}\ldots \upsilon_{n-1}\sigma_{n}l_{n}. \end{array}$$

This lemma is easily proved by the basic determinant transformations and can be justified by some special cases. For example, we consider the case σ_*i*_ = 0, *i* = 1,…,*n*. It is noticed that at this time, matrix *A* is a diagonal matrix, then we have that det[*A*] = υ_1_υ_2_,…,υ_*n*_, which accords with the conclusion obtained from Lemma 1. Also, it can be seen that det[*A* − μI] can be directly computed by Lemma 1 (where I is a unit matrix). Hence, the eigenvalues of matrix *A* can be solved by this Lemma.

In model (9), we may assume that *k* = 1,2,…,*n* since we consider a finite population.^18^ Upon omitting higher powers of ρ_*k*_, we can get the linear differential equations

$$\frac{d}{\mathrm{dt}}\rho_{k}(t)=-\rho_{k}+\lambda (k-\alpha )\psi (k)\Theta ,$$

which implies that the Jacobian matrix of Eq. (9) is

$$J_{0}=\left[\begin{array}{ccccc} \sigma_{1}l_{1}-1 & \sigma_{1}l_{2} & \sigma_{1}l_{3} & \ldots & \sigma_{1}l_{n}\\ \sigma_{2}l_{1} & \sigma_{2}l_{2}-1 & \sigma_{2}l_{3} & \ldots & \sigma_{2}l_{n}\\ \sigma_{3}l_{1} & \sigma_{3}l_{2} & \sigma_{3}l_{3}-1 & \ldots & \sigma_{3}l_{n}\\ \ldots & \ldots & \ldots & \ldots & \\ \sigma_{n}p_{1} & \sigma_{n}l_{2} & \sigma_{n}l_{3} & \ldots & \sigma_{n}l_{n}-1 \end{array}\right],$$

where σ_*k*_:= λ (*k* − α)ψ(*k*) and *l*_*k*_:= *kP*(*k*)/〈*k*〉.

Obviously, the local stability of the infection-free equilibrium is determined by the stability of matrix *J*_0_. We now compute the eigenvalues of matrix *J*_0_ by Lemma 1. Let, *J*_0_ − μI = *M* = (*m*_*ij*_). If we define σ_*k*_,*l*_*k*_ as stated above and *v*_*k*_ = −1 − μ, then *m*_*ij*_ = δ_*ij*_υ_*i*_ + σ_*i*_*l*_*j*_. According to Lemma 1, we have

$$\text{det}[J_{0}-\mu I]={\left(-1-\mu \right)}^{n-1}\left(-1-\mu +\sum_{k=1}^{n}\sigma_{k}l_{k}\right).$$

Upon solving equation det[*J*_0_ − μI] = 0, one can obtain *n* eigenvalues: *n* − 1 eigenvalues equal to −1 (that is, μ_1_ = ··· = μ_*n* − 1_ = −1) and the *n*th eigenvalue

$$\mu_{n}=\sum_{k=1}^{n}\sigma_{k}l_{k}-1.$$

Apparently, μ_*n*_ is the maximal eigenvalue. So the infection-free equilibrium is locally stable if and only if μ_*n*_ < 0 which leads to

$$\lambda >\lambda_{c}=\frac{〈k〉}{〈k^{2}\psi (k)〉-\alpha 〈k\psi (k)〉}.$$ (11)

This shows that the dependence of an epidemic outbreak on both contact awareness and local awareness, while global awareness has no influence whatsoever.

## IV. SIMULATIONS

In Sec. III, we obtained the condition for an epidemic outbreak under the three forms of awareness. We know that both the contact awareness and the local awareness play an important role in determining whether an infectious disease prevails in a population. On the other hand, the epidemic threshold is independent of the global awareness. In this section, we demonstrate these theoretical results using Monte-Carlo stochastic simulations (SS).

**FIG. 1.**
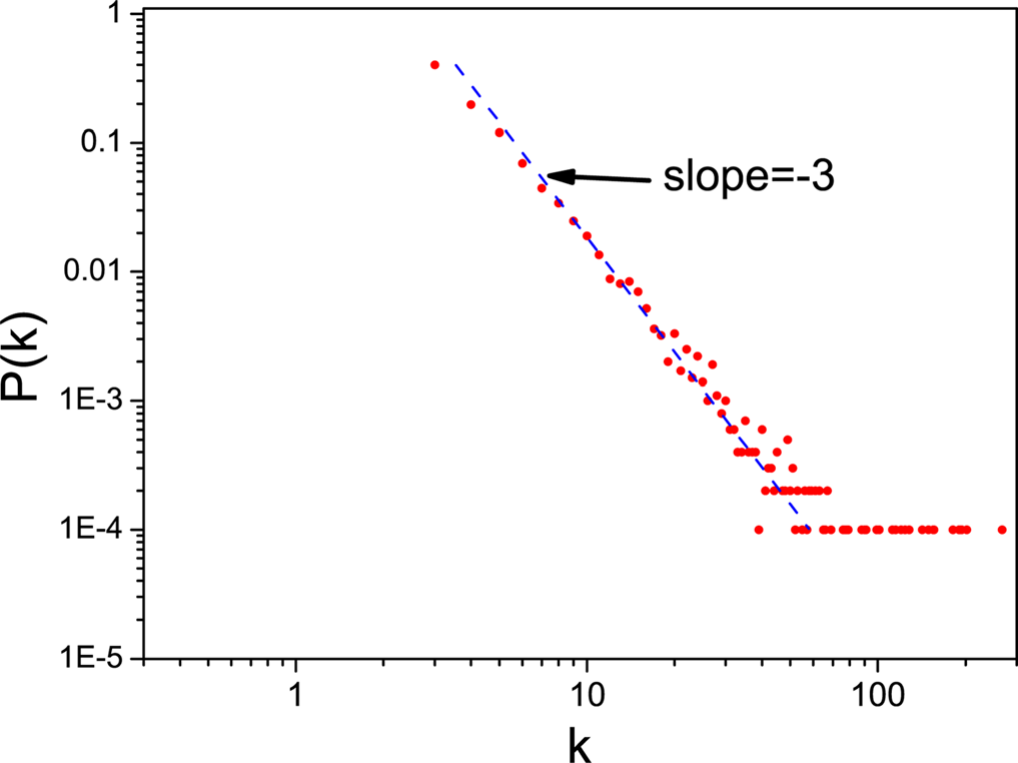
(Color online) The degree distribution of a BA scale-free network used in our simulations. This plot shows that *P*(*k*) ∼ *k*^−3^.

Simulations of SIS dynamics are performed using a Barabási-Albert (BA) scale-free network^17^ with the degree distribution *P*(*k*) ∼ *k*^−3^ (see Fig. 1) and the network size *N* = 10 000. All simulations begin with the initial state where 1% of the nodes are infected and iterate the rules of the SIS model with parallel updating until convergence to a steady state, either absorbing or active. The SIS dynamics are totally evolved for 1000 time steps. As the steady state is a dynamical equilibrium, we make time average to reduce the fluctuation of ρ(*t*). So, we let $\rho =\frac{1}{T}\sum_{t=t_{0}}^{t_{0}-1+T}\rho (t)$ and take *T* = 50 (that is, *t*_0_ = 951). To minimise random fluctuation caused by the initial conditions, we make average of ρ over 50 realizations of different initial infectious nodes.

In addition, since ψ(*k*) is a decreasing function of *k*, we consider the contact awareness with a form ψ(*k*) = *k*^−*b*^, where *b* ≥ 0. Upon substituting it into Eq. (11), we have

$$\lambda_{c}=\frac{〈k〉}{〈k^{2-b}〉-\alpha 〈k^{1-b}〉}.$$ (12)

We mainly examine the dependence of λ_*c*_ on the parameters α and β. In the network with a broad distribution, the ratio 〈*k*^2^〉/〈*k*〉 is very large.^26^ Hence, when *b* = 0, the effect induced by the local awareness is very small. In order to observe the relation between the epidemic threshold λ_*c*_ and parameters α, β clearly, we consider the two scaling schemes: *b* = 0.3 and *b* = 0.8.

We first consider the case *b* = 0.3. In this case, 〈*k*^2−*b*^〉/〈*k*〉 is still very large and the impact of the fluctuation of the degree distribution on the epidemic threshold is strong. The epidemic threshold λ_*c*_ in stochastic simulations is measured by the following way. Let, λ increase systematically by 0.01 in the interval [0, 1] and we compute ρ for each λ. When ρ > 0.0005 at λ_1_, we set λ_*c*_ = λ_1_ − 0.01.

**FIG. 2.**
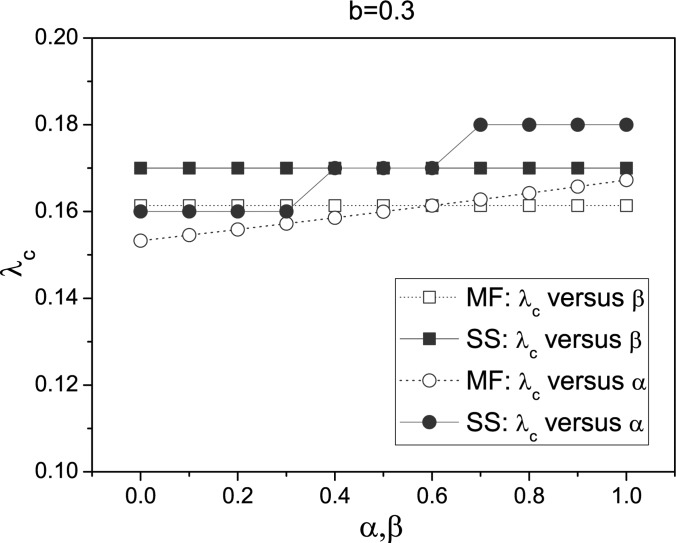
(Color online) Plot of λ_*c*_ versus α and β with ψ(*k*) = *k*^−0.3^. When considering λ_*c*_ versus α, we set β = 0.3; when considering λ_*c*_ versus β, we set α = 0.6. “SS” means stochastic simulations and “MF” means mean-field predictions. All stochastic simulations are performed on the same BA scale-free networks and mean-field predictions are obtained by numerically integrating the ordinary differential Eq. (9), where the degree distribution *P*(*k*) is obtained from the stochastic simulation.

In Fig. 2, we illustrate the change of λ_*c*_ with respect to α and β both for stochastic simulations (for short, SS denoted by solid symbol) and also for mean-field (MF, denoted by open symbol) predictions Eq. (12). It is clear that the epidemic threshold λ_*c*_ is unchanged for different β; while, it increases with α. These results are in accordance with the mean-field prediction Eq. (12). The discrepancy between these can also be shown in our simulations. We can see that the simulation results are slightly larger than the expected values obtained from Eq. (12), which is likely to be due to a distribution cutoff effect on a finite size network.^23^

**FIG. 3.**
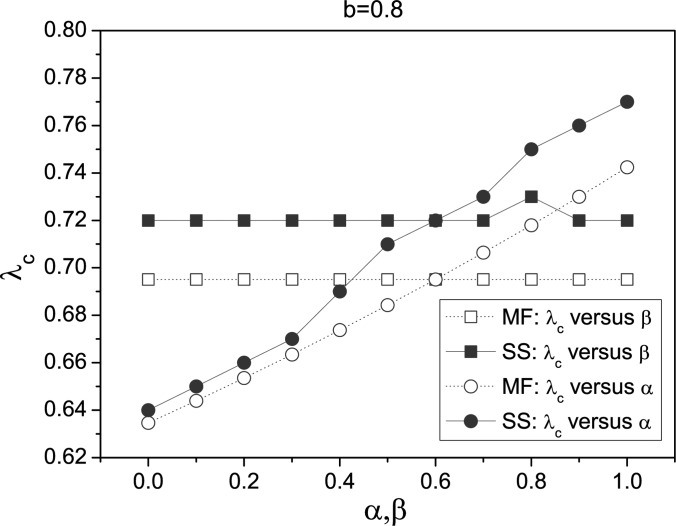
(Color online) Plot of λ_*c*_ versus α and β with ψ(*k*) = *k*^−0.8^. When considering λ_*c*_ versus α, we set β = 0.3; when considering λ_*c*_ versus β, we set α = 0.6. All the simulations are performed on the same BA scale-free networks as illustrated in Fig. 2.

Next, we consider the case *b* = 0.8. This is also a typical case, which represents for the weak impact of the fluctuation of the degree distribution. According to our simulations in Fig. 3, we also find that λ_*c*_ is almost unchanged for different β; while, it still increases with α. The difference with the case *b* = 0.3 is that the epidemic threshold has a broad range. This phenomenon indicates the influence degree of local awareness on the epidemic threshold is related with the contact awareness. The contact awareness seems to facilitate the effect of local awareness on the epidemic threshold.

**FIG. 4.**
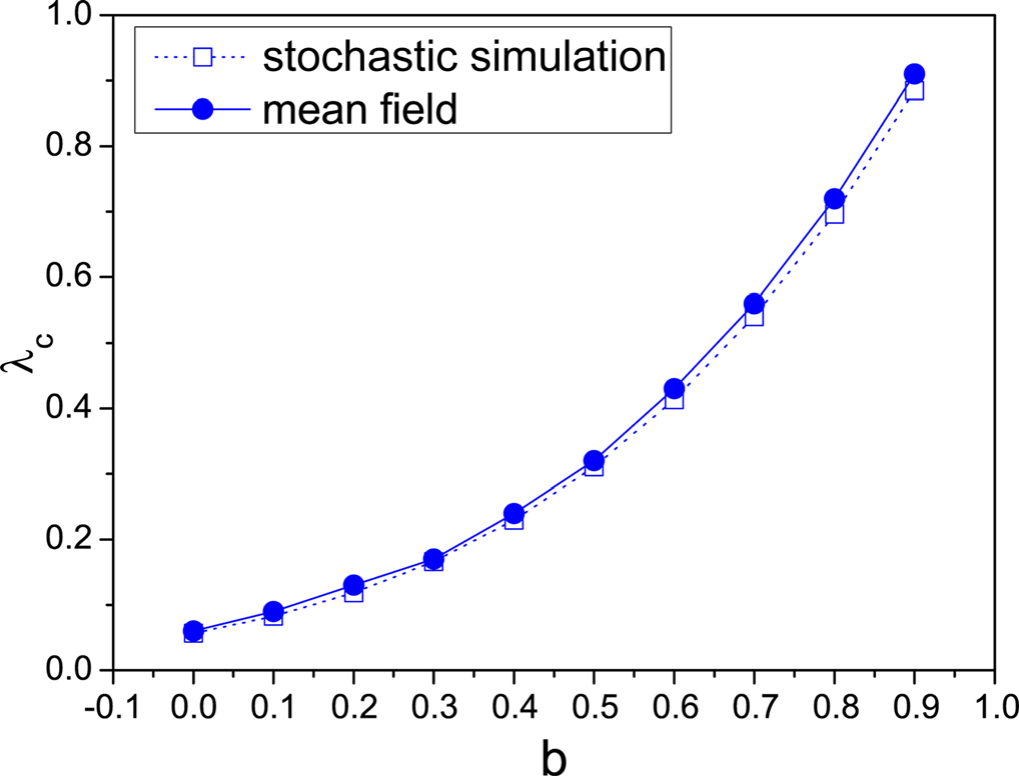
(Color online) Plot of λ_*c*_ versus *b*. We use parameters α = 0.6 and β = 0.3. All the simulations are performed on the same BA scale-free networks as illustrated in Fig. 2.

From Figs. 2 and 3, one can see that the scaling scheme *b* has significant effect on the value of λ_*c*_. We also investigate the change of λ_*c*_ with *b* in Fig. 4 under α = 0.6 and β = 0.3. In this plot, we do not find the epidemic threshold λ_*c*_ corresponding to the case *b* = 1. This is the reason that λ_*c*_ = 〈*k*〉/(〈*k*〉 − 0.6) > 1, which exceeds the range of λ. All these results show that simulations agree well with theoretical predictions.

**FIG. 5.**
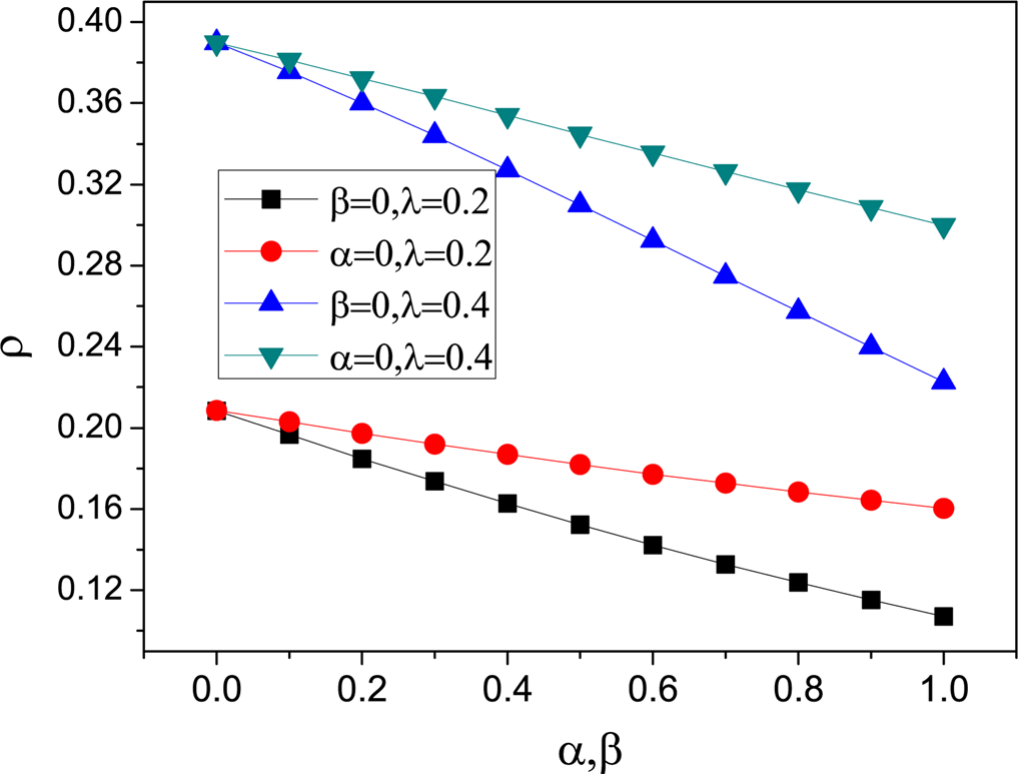
(Color online) The effect of parameter α and β on the final epidemic size ρ for λ = 0.2 and λ = 0.4 under *b* = 0. All the simulations are performed on the same BA scale-free networks.

The threshold formula Eq. (11) clearly shows us that the local awareness has stronger impact on disease dynamics than the global awareness. Although the global awareness has no effect on the epidemic threshold and one cannot decrease the likelihood of an epidemic outbreak through increasing the global awareness (or β), it can decrease the epidemic prevalence. This is in accordance with the previous result^9^ and can be verified by simulations. Simulations in Fig. 5 shows that the final epidemic size ρ decreases with β regardless of λ = 0.2 or λ = 0.4. Fig. 5 also shows that ρ decreases with α. In general, the rate of change of final epidemic size with respect to α, $\frac{\partial \rho }{\partial \alpha }\leq 0$ and the rate of change of final epidemic size with respect to β, $\frac{\partial \rho }{\partial \beta }\leq 0$.

We further find that the profiles in Fig. 5 are almost straight lines and for the same λ the slope of line ρ vs α is smaller than one of line ρ vs β, which can be clearly observed since the two lines go across the same point (at this case, α = β = 0). So, one can get that the impact of the local awareness on the epidemic prevalence is more stronger in our model. In order to completely investigate the discrepancy between the local awareness and the global awareness about their influence degrees on the epidemic prevalence, we would like to propose a quantity to characterize this. Such quantity is defined as follows:

$$\Delta F:=\frac{\partial \rho }{\partial \alpha }-\frac{\partial \rho }{\partial \beta },$$ (13)

which is a simple subtraction of two rates of change of final epidemic size. Since $\frac{\partial \rho }{\partial \alpha }\leq 0$ and $\frac{\partial \rho }{\partial \beta }\leq 0$, the inequality Δ*F* < 0 shows that the impact of local awareness/information is greater; otherwise, Δ*F* > 0 indicates that the impact of global awareness is greater. As an illustration, from Fig. 5, we can find that Δ*F* < 0 when α = β = 0.

In order to estimate the value of Δ*F* in stochastic simulations, we take an approximate calculation

$$\Delta F(\alpha ,\beta )≃\frac{1}{ɛ}[\rho (\alpha +ɛ,\beta )-\rho (\alpha ,\beta +ɛ)].$$

**FIG. 6.**
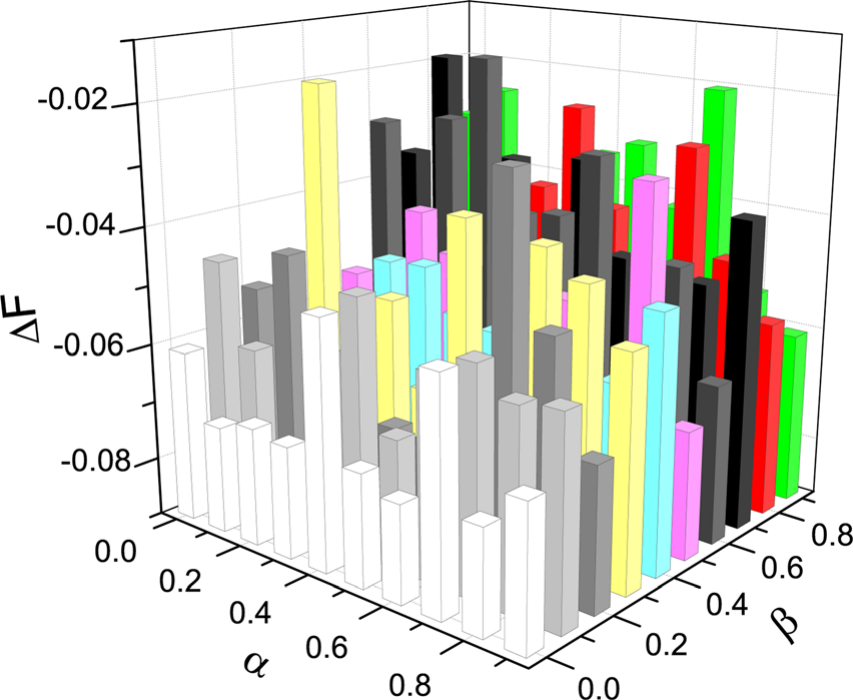
(Color online) The variation of Δ*F* with respect to α and β. Parameters: ɛ = 0.01, λ = 0.2 and *b* = 0.

From Fig. 6, one can observe the range of variation of Δ*F* with respect to two parameters (α, β) in the model. Through this simulation, we confirm that Δ*F* < 0 and find its absolute value |Δ*F*| > 0.01. These tell us that the local awareness has a stronger impact on the epidemic prevalence than the global awareness.

In the final part of this section, we examine the accuracy of model (9) for prediction of the stationary prevalence. To this end, we performed one thousands of stochastic simulations, in which λ is replaced with λ*h* and γ is replaced with γ*h*.

**FIG. 7.**
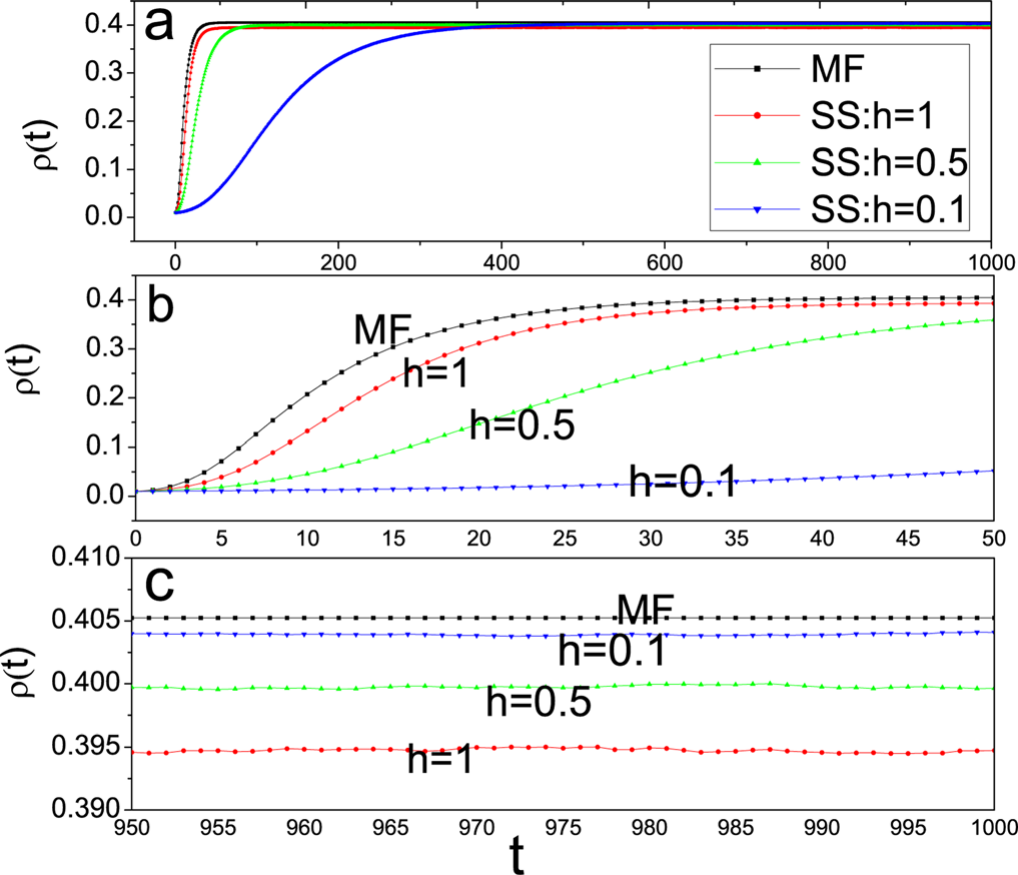
(Color online) Comparison of a mean-field prediction Eq. (9) and the average of 1000 runs of stochastic simulations for the SIS model on the same BA scale-free network with α = 0.6, β = 0.3, λ= 0.05, γ = 0.1, *b* = 0, *N* = 10 000, 〈*k*〉 = 6. In stochastic simulations, we take *h* = 0.1, *h* = 0.5, and *h* = 1, respectively. This plot displays different time ranges: (a) *t* ∈ [0,1000]; (b) *t* ∈ [0,50]; (c) *t* ∈ [950,1000].

Fig. 7 shows there is a small discrepancy between the mean-field theory and stochastic simulations. Stochastic simulations are consistently lower than mean field calculations (see Figs. 7(a) and 7(b)). As we know, the smaller (larger) the value of λ_*c*_(ρ), the more serious the epidemic disease. Hence, this is consistent to the results shown in Figs. 2, 3, and 4. In addition, we also see that the mean-field approach is still efficient, especially for small *h* (see Fig. 7(c)).

## V. CONCLUSIONS AND DISCUSSIONS

We have presented an analytical framework for studying the impact of three forms of epidemiological awareness on disease dynamics, i.e., contact awareness which increases with individual contact number, local awareness which increases with the fraction of infected contacts, and global awareness which increases with the overall disease prevalence. All three forms of awareness can reduce susceptibility to infection. Theoretical analysis and computational simulations indicate that both the contact awareness and the local awareness can raise the epidemic threshold to control epidemic outbreak, while the global awareness only decrease the epidemic prevalence. Hence, even in the absence of immunization procedures or quarantine/isolation measures, an epidemic disease can be controlled by human adaptive reactions.^7,9^ These results accord with previous findings.^5,9^

It is interesting to explore one particular problem: how can the local information have such a strong effect on disease dynamics compared to the global information under the same conditions?^4^ Why can the local awareness raise the epidemic threshold but not the global awareness? We attempt to give a possible illustration. We think this is closely related with the heterogeneity of information for the following reasons.

**FIG. 8.**
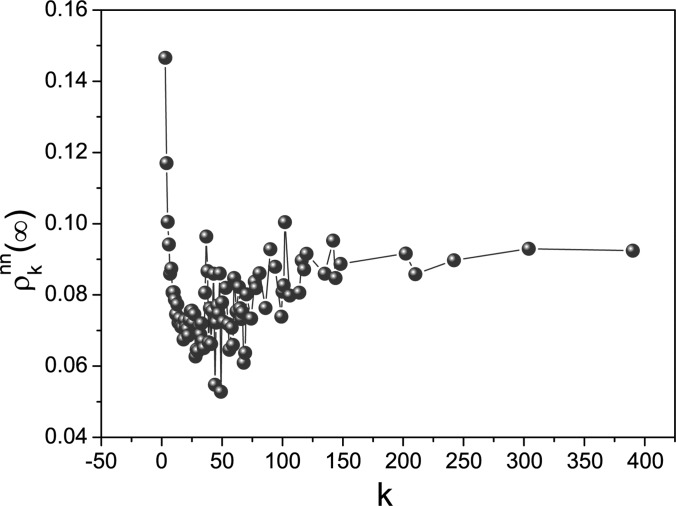
(Color online) The plot of the local infection density at the steady state $\rho_{k}^{\mathrm{nm}}(\infty )$ with respect to *k*. Parameters: λ = 0.1, *b* = 0, α = 0.6, and β = 0.3.

If we only consider the global information, it is easy to see that these are identical to each other in our model since *x* = ρ is not dependent of node in a population. However, it is not the case for the local information. Let us make stochastic simulations to show this. We consider the averaged infection fraction in the nearest neighborhood (NN) of node *i* with degree *k* at the steady state, denoted by $\rho_{k}^{\mathrm{nm}}(\infty )$. In Fig. 8, the relation between $\rho_{k}^{\mathrm{nm}}(\infty )$ and *k* is numerically investigated. This plot clearly illustrates the obvious difference from $\rho_{k}^{\mathrm{nm}}(\infty )$ with respect to *k*, and further tells us that $\varrho_{i}$ as a function of node *i* is not uniform. Consequently, for all nodes in a population, the global information is homogenous but the local information is heterogenous (this is similar to the effect of contact awareness^23^). The heterogeneity of information leads to the heterogeneity of individual awareness. This further leads to the heterogeneity of the infection rate owing to the definition (1). As we know,^22,23^ heterogenous infection rates potentially stop an epidemic outbreak.

In the present paper, we adopted a prompt information reaction mechanism, as an approximation to reality. Nevertheless, from real viewpoints, the information reaction should be of slowness or retardation for an individual. In our model, the epidemic model does not display oscillatory behavior.^14^ However, if we consider the slow or retarded reactions of awareness, the case would be different.^33^ Hence, one may consider other information updating mechanisms, e.g., periodic updating or delayed updating. Also, it is interesting to study the impact of awareness on the epidemic spreading in mobile populations.^34,35^

## APPENDIX: PROOF OF Eq. (8)

In this appendix, we give the detailed proof of Eq. (8) in Sec. II.

Proof of Eq. (8): Let $H(s,k)=1-\lambda h\psi (k)(1-\frac{\alpha s}{k})\left(1-\beta \rho \right)$, then we have

$$\begin{array}{cc} & \sum_{s}B(k,s){\left[1-\lambda h\psi (k)\left(1-\frac{\alpha s}{k}-\beta \rho \right)+o(h)\right]}^{s}\\ & =\sum_{s}B(k,s){\left[H(s,k)+o(h)\right]}^{s}\\ & =\sum_{s}B(k,s)\left[\left(\begin{array}{c} s\\ 0 \end{array}\right)H^{s}+\left(\begin{array}{c} s\\ 1 \end{array}\right)H^{s-1}o(h)+\ldots \left(\begin{array}{c} s\\ s \end{array}\right)o^{s}(h)\right]\\ & =\sum_{s}B(k,s)H^{s}+o(h). \end{array}$$

Based on the above result, it is easy to get Eq. (8).
